# Aetiological pathways to Borderline Personality Disorder symptoms in early adolescence: childhood dysregulated behaviour, maladaptive parenting and bully victimisation

**DOI:** 10.1186/s40479-017-0060-x

**Published:** 2017-06-03

**Authors:** Catherine Winsper, James Hall, Vicky Y. Strauss, Dieter Wolke

**Affiliations:** 1 Division of Mental Health and Wellbeing, Warwick Medical School, University of Warwick, Coventry, CV4 7AL UK; 2 University of Exeter, Exeter, UK; 3 Centre for Statistics in Medicine, University of Oxford, Oxford, UK; 4 Department of Psychology, University of Warwick, Coventry, UK

**Keywords:** BPD, Dysregulated behaviour, Bullying, Harsh parenting, Structural equation modelling, ALSPAC

## Abstract

**Background:**

Developmental theories for the aetiology of Borderline Personality Disorder (BPD) suggest that both individual features (e.g., childhood dysregulated behaviour) and negative environmental experiences (e.g., maladaptive parenting, peer victimisation) may lead to the development of BPD symptoms during adolescence. Few prospective studies have examined potential aetiological pathways involving these two factors.

**Method:**

We addressed this gap in the literature using data from the Avon Longitudinal Study of Parents and Children (ALSPAC). We assessed mother-reported childhood dysregulated behaviour at 4, 7 and 8 years using the Strengths and Difficulties Questionnaire (SDQ); maladaptive parenting (maternal hitting, punishment, and hostility) at 8 to 9 years; and bully victimisation (child and mother report) at 8, 9 and 10 years. BPD symptoms were assessed at 11 years using the UK Childhood Interview for DSM-IV BPD. Control variables included adolescent depression (assessed with the Short Moods and Feelings Questionnaire-SMFQ) and psychotic symptoms (assessed with the Psychosis-Like Symptoms Interview-PLIKS) at 11 to 14 years, and mother’s exposure to family adversity during pregnancy (assessed with the Family Adversity Scale-FAI).

**Results:**

In unadjusted logistic regression analyses, childhood dysregulated behaviour and all environmental risk factors (i.e., family adversity, maladaptive parenting, and bully victimisation) were significantly associated with BPD symptoms at 11 years. Within structural equation modelling controlling for all associations simultaneously, family adversity and male sex significantly predicted dysregulated behaviour across childhood, while bully victimisation significantly predicted BPD, depression, and psychotic symptoms. Children displaying dysregulated behaviour across childhood were significantly more likely to experience maladaptive parenting (β = 0.075, *p* < 0.001) and bully victimisation (β = 0.327, *p* < 0.001). Further, there was a significant indirect association between childhood dysregulated behaviour and BPD symptoms via an increased risk of bullying (β = 0.097, *p* < 0.001). While significant indirect associations between dysregulated behaviour, bully victimisation and depression (β = 0.063, *p* < 0.001) and psychotic (β = 0.074, *p* < 0.001) outcomes were also observed, the indirect association was significantly stronger for the BPD outcome (BPD – depression = 0.034, *p* < 0.01; BPD – psychotic symptoms = 0.023, *p* < 0.01).

**Conclusions:**

Childhood dysregulated behaviour is associated with BPD in early adolescence via an increased risk of bully victimisation. This suggests that childhood dysregulation may influence the risk of bully victimisation, which in turn influences the development of BPD. Effective interventions should target dysregulated behaviour early on to reduce exposure to environmental risks and the subsequent development of BPD.

## Background

Borderline personality disorder (BPD) is a serious mental illness associated with suicidal behaviour, severe behavioural and emotional dysregulation, high rates of comorbid mental disorder, and great costs to society [1, 2]. BPD diagnosis in childhood and adolescence remains a controversial topic [3, 4]. Nevertheless, BPD is unlikely to appear *de novo* in early adulthood, but rather may be considered as the endpoint following the emergence of precursor BPD symptoms during childhood or early adolescence [5–7]. Importantly, the identification of BPD symptoms prior to adulthood may help shed light on aetiological processes [5], inform early intervention programs [8], and ensure youth with personality problems receive appropriate treatment [9].

### Developmental theories for the aetiology of BPD

Extant theories for the aetiology of BPD hypothesise a stress-diathesis model in which BPD represents an endpoint following a series of complex interactions between biological factors and environmental influences [10].

In particular, disturbed relationships are highlighted as a potential endophenotype for BPD [11]. Indeed, troubled interactions (e.g., maladaptive parenting, bullying) likely represent a central process in the development of BPD, and along with the child’s own behavioural and emotional vulnerabilities, are proposed to increase risk. Aetiological models suggest that the developing child may exhibit behaviours which have a direct impact on the social or family environment [10, 12]. For example, dysregulated behaviour in childhood could elicit negative reactions from both parents, e.g., lack of sensitivity [13], and peers, e.g., bullying [14]. These reactions, in turn, could subsequently increase risk for BPD [15, 16].

There are few studies that have prospectively examined the combined effects of individual and environmental risk factors on BPD. Belsky, Caspi [17] reported that inherited diathesis interacted with environmental stress during childhood to increase the risk of BPD symptoms at 12 years, i.e., children who experienced harsh parenting between 5 and 10 years were at greatly increased risk of developing BPD symptoms if they also had a positive family history of psychiatric illness. In a shorter-term prospective study of 11 to 13 year olds, Jovev, McKenzie [18] reported that abuse (physical, sexual or emotional) acted as a moderator of the effect of temperament (i.e., low affiliation) on BPD symptoms assessed 2 years later. In the most recent study, Stepp, Whalen [19] examined transactions between BPD symptoms and parenting practices over 4 years (age 14 to 17). In a large community sample of adolescent girls, they demonstrated that the developmental trajectories of BPD symptoms and parenting (harsh punishment and low caregiver warmth) were moderately associated. This suggests that there is a reciprocal relationship between parenting experiences and BPD symptoms.

While these important studies provide some prospective evidence for individual-environmental interactions in the development of BPD during adolescence, they only included parent-child interactions as indicators of environmental risk. Furthermore, with the exception of Belsky, Caspi [17], development was considered over a relatively short period of time during adolescence, thus early childhood precursors were omitted from the analysis. Finally, studies focused on interactional rather than mediational associations. Thus, we cannot draw conclusions regarding the mechanisms underpinning associations between individual features, environmental risk factors, and subsequent BPD.

Youngsters spend increasing amounts of time with their peers (and correspondingly less time with their families) as they progress through childhood into adolescence [20]. Subsequently, problematic peer interactions may represent an important, developmentally salient risk for borderline symptomatology in adolescence [21, 22]. Indeed, recent studies have highlighted bullying experiences during childhood as a potential risk factor for BPD in both adult [23] and adolescent [15] populations. In a study using the ALSPAC cohort, Wolke, Schreier [15] reported that child reported chronic bullying led to a five times increased odds (OR: 5.44; 95% CI: 3.86–7.66) of 5 or more BPD symptoms. This suggests that a more comprehensive test of the combined effects of individual features and environmental risk should incorporate assessments of peer-child, in addition to parent-child, transactions.

### The childhood dysregulation phenotype

As described above, individual characteristics of the child, such as dysregulated behaviour, may elicit reactions from the environment that could potentiate risk for BPD. Of late there has been increasing interest in the *childhood dysregulation phenotype*, which encompasses an assessment of emotional, behavioural and cognitive dysregulation. This behavioural phenotype has been shown to be highly heritable [24, 25]; stable over time [26–28]; and strongly associated with prior infant and toddler regulatory problems [27]. Of note, the childhood dysregulation phenotype predicts a range of psychiatric problems including personality disorder traits in adolescence/early adulthood [25, 29]. It is therefore plausible that the childhood dysregulation phenotype (via its combined effects with environmental risks) may represent a salient risk indicator for the early development of BPD [10].

## The current study

In the current study, we addressed the existing gaps in the literature by considering potential aetiological pathways involving childhood dysregulated behaviour at 4–8 years, negative interactions with peers and parents at 8–10 years, and BPD symptoms at 11 years. We conducted path analyses to enable us to examine direct and indirect (mediational) associations between dysregulated behaviour, environmental risks, and BPD.

Specifically, we sought to elucidate whether dysregulated behaviour across childhood increased the risk of negative social interactions, subsequently increasing risk of BPD symptoms.

## Method

### Sample description

The Avon Longitudinal Study of Parents and Children (ALSPAC) enrolled 14, 541 women resident in the English region of Avon if they had an expected delivery date between 1st April 1991 and 31st December 1992. A total of 13, 971 children formed the original cohort. From the first trimester of pregnancy, parents completed postal questionnaires about themselves, and the study child’s health and development. Please note that the study website contains details of all the data that is available through a fully searchable data dictionary (see http://www.bristol.ac.uk/alspac/researchers/data-access/data dictionary/).

Children were invited to attend annual assessment clinics, including face-to-face interviews, and psychological and physical tests from 7 years onwards [30]. Of the original 13, 971 children, 7159 attended the assessment clinic with BPD interview. We included data from those who completed at least eight of the nine sections of the BPD interview (6, 050), as a small number of children responded “don’t know” to some of the BPD items. Inclusion in the final sample was also dependent on the child having depression and psychotic symptom measures at age 11 to 14 years. A total of 4, 826 (34.5% of the original cohort) cases met these inclusion criteria. Those excluded were more often male, exposed to more family adversity, had significantly higher childhood dysregulated scores at 4, 7 and 8 years, were more often punished, and were more often victims of bullying (see Table 1). Ethical approval for the study was obtained from the ALSPAC Ethics and Law committee and the local research ethics committee.

**Table 1:** Drop-out analysis comparing those included in the analysis to those lost to attrition

Characteristic	Included (% or mean; sd)	Not included (% or mean; sd)	Included as reference category Odds Ratio (95% CI)
Gender			
Male	2324 (32.2%)	4896 (67.8%)	[reference]
Female	2502 (37.0%)	4254 (63.0%)	**0.81 (0.75, 0.87)**
Family adversity	0.99 (1.35)	1.51 (1.71)	**1.26 (1.22. 1.29)**
Dysregulated behaviour 4 years	7.00 (3.74)	7.70 (4.00)	**1.05 (1.04, 1.06)**
Dysregulated behaviour 7 years	6.18 (4.13)	6.89 (4.55)	**1.04 (1.03, 1.05)**
Dysregulated behaviour 8 years	6.18 (3.92)	6.82 (4.27)	**1.04 (1.03, 1.05)**
Maternal hitting	0.61 (0.63)	0.64 (0.68)	1.07 (0.99, 1,15)
Maternal punishment	1.68 (0.98)	1.73 (1.03)	**1.05 (1.00, 1.10)**
Maternal hostility	0.88 (1.04)	0.91 (1.04)	1.03 (0.98, 1.07)
Bully victimisation at 8			
No	2651 (65%)	1426 (35%)	[reference]
Yes	1642 (60%)	1096 (40%)	**1.03 (1.02, 1.04)**
Bully victimisation at 9			
No	3382 (58.3%)	2421 (41.7%)	[reference]
Somewhat true	814 (54.7%)	764 (45.3%)	**1.13 (1.00, 1.23)**
Certainly true	108 (50.0%)	108 (50.0%)	**1.40 (1.07, 1.83)**
Bully victimisation at 10			
No	3569 (67.5%)	1717 (32.5%)	[reference]
Yes	1095 (62.9%)	645 (37.1%)	**1.03 (1.02, 1.05)**

Boldface indicates a significant association

### Measures

#### Symptoms of borderline personality disorder

Borderline personality symptoms were assessed using a face-to-face semi-structured interview: the UK Childhood Interview for DSM-IV Borderline Personality Disorder [UK-CI-BPD] [31]. The UK-CI-BPD is based on the borderline module of the Diagnostic Interview for DSM-IV Personality Disorders [32], which is a widely used semi-structured interview for all DSM-IV Axis II disorders. The inter-rater and test–retest reliability of the DSM-III, DSM-III-R and DSM-IV versions of this measure have all proven to be good to excellent [33, 34]. The UK-CI-BPD was adapted from the CI-BPD (US version). The convergent validity of the CI-BPD was investigated using 171 adolescents aged 13–17 years; 111 met criteria for BPD and 60 were normal comparison subjects. A Spearman’s r of 0.89 was obtained when comparing a dimensional score for BPD on the CI-BPD with the total score on the Revised Diagnostic Interview for Borderlines. The inter-rater reliability (k) of the UK-CI-BPD assessed from taped interviews of 30 children ranged from 0.36 to 1.0 (median value 0.88). 86% of the k values were within the excellent range of >0.75 [35].

The UK-CI-BPD is the first semi-structured interview assessing DSM-IV BPD in children and adolescents. Similar to DSM-IV criteria, the interview consists of nine sections: intense inappropriate anger; affective instability; emptiness; identity disturbance; paranoid ideation; abandonment; suicidal or self-mutilating behaviours; impulsivity and intense unstable relationships. Once a trained assessor had explored each section, a judgment was made as to whether each symptom was definitely present, probably present, or absent. A symptom was classed as definitely present if it occurred daily or approximately 25% of the time, and probably present if it had occurred repeatedly, but did not meet the criterion for definitely present. The derived dichotomous outcome was based on previous studies [15, 36, 37] and represented the frequent (daily/25% of the time) or repeated occurrence of five or more BPD symptoms. Although BPD is sometimes measured dimensionally in young populations [22], we elected to use a dichotomous outcome because we were interested in assessing associations with BPD symptoms crossing the established clinical threshold (i.e., 5 or more symptoms). However, it should be noted that a diagnosis of BPD according to the diagnostic statistical manual is based on the presence of five or more definite features, making our assessment more sensitive.

### Alternative psychopathologies: depression and psychotic symptoms

Depression symptoms were assessed using the Short Moods and Feelings Questionnaire (SMFQ): a 13-item scale measuring depression symptoms, demonstrating high reliability and validity [38]. The child completed the SMFQ at 11 & 14 years during assessment clinics. The mother responded via postal questionnaire when the child was 12 & 13 years. Each item is rated on a 3-point scale referring to events occurring in the past two weeks. In line with previous studies we constructed a dichotomous depression variable to represent severe depression symptoms, i.e., those occurring within a clinical range [39]. Scores from each time-point were standardised, and depression symptoms were classed as present if the child was in the top 90th percentile during either the early (11–12 years) or late (13–14 years) assessment period.

Children were asked about their psychotic symptoms using the Psychosis-like Symptoms Interview [40] when they were 12 years of age. Using 12 stem questions, psychology graduates rated whether adolescents had experienced any hallucinations, delusions or thought disorders in the previous 6 months. Consistent with previous studies, a dichotomous psychotic outcome variable was derived according to the definite or suspected presence of 1 or more psychotic symptoms [41, 42].

### Childhood dysregulation

Mothers completed the Strengths and Difficulties Questionnaire [SDQ] [43] when the child was 4, 7 and 8 years old. Item response was scaled from 0 to 2, with 0 corresponding to “not true”; 1 corresponding to “somewhat true” and 2 corresponding to “certainly true.” Responses from the three subscales (5 items per subscale): *negative emotionality* (e.g., child has many worries), *conduct disorder* (e.g., child steals from home) and *hyperactivity* (e.g.*, child is easily distracted*) were summed to derive a total childhood dysregulation score (scale of 0 to 30) for each child at each time point. These three scores were included in the path analysis as indicators to model a latent childhood dysregulated behaviour factor [36].

### Environmental risk factors: bullying and maladaptive parenting

Bully victimisation was assessed at 8, 9 and 10 years. Child report was derived from the Bullying and Friendship Interview Schedule [42] at 8 and 10 years. Consistent with previous work [15], we constructed a bully victimisation severity variable. We summed the four relational bullying items, e.g., “spreading lies about child” (scale 0–3) and the five overt bullying items, e.g., “having belongings stolen” (scale 0–3). Thus, the severity scale could range from 0 to 27 for each time point (8 and 10 years). Mothers reported on their child’s experience of bully victimisation at 9 years with the following responses: 0 = no bullying; 1 = child bullied is “somewhat true;” 2 = child bullied is “certainly true.” We used the three bullying variables (i.e., at 8, 9, and 10 years) as indicators in the path analysis to create a latent bully victimisation factor [36].

Maladaptive parenting was assessed when the child was 8 to 9 years of age, using three indicators: maternal hitting (9 years), punishment (9 years), and hostility (8 years). Mothers’ responses to “child is hit” and “child is punished” were as follows: Never = 0; rarely =1; once or twice a month =2; once or twice a week = 3; several times a week = 4; and every day = 5. Hostility was ascertained by three items: “mother often gets irritated by child,” “mother has frequent battle of wills with study child,” and “child gets on mothers nerves,” which were summed to create a variable ranging from 0 to 3 [44]. We used these three variables (i.e., hitting, punishment, and hostility) as indicators in the path analysis to create a latent maladaptive parenting factor [36].

### Confounding factors

Gender (51.5% girls) was included in the path analysis due to the observed male bias in self-control problems [45]. Family adversity was incorporated into the path analysis due to associations between psychosocial adversity and childhood dysregulated behaviour [46] and psychopathology [47]. Mothers were questioned about their exposure to multiple family risk factors during pregnancy using the Family Adversity Index (FAI). The index consists of 18 items including: housing problems, financial difficulties, maternal affective disorder, substance abuse, and involvement in crime [47].

### Data analytic plan

#### Logistic regression analysis

Using *SPSS version 22*, we conducted unadjusted logistic regressions to examine whether childhood dysregulated behaviour and environmental risk factors were associated with BPD symptoms at 11 years. Results are reported as Odds Ratios (OR) with 95% Confidence Intervals (CIs).

### Structural Equation Modelling (SEM) to examine the direct and indirect (via environmental risks) pathways from childhood dysregulation to BPD symptoms

SEM was conducted using *Mplus version 6* to assess the direct and indirect (i.e., mediated) associations between childhood dysregulated behaviour, environmental risk factors and BPD symptoms at 11 years. We conducted the SEM in two stages. First, we constructed our Confirmatory Factor Analysis (CFA) model for the latent variables (i.e., dysregulated behaviour, maladaptive parenting, and bully victimisation) to test the measurement model.

**Fig. 1:**
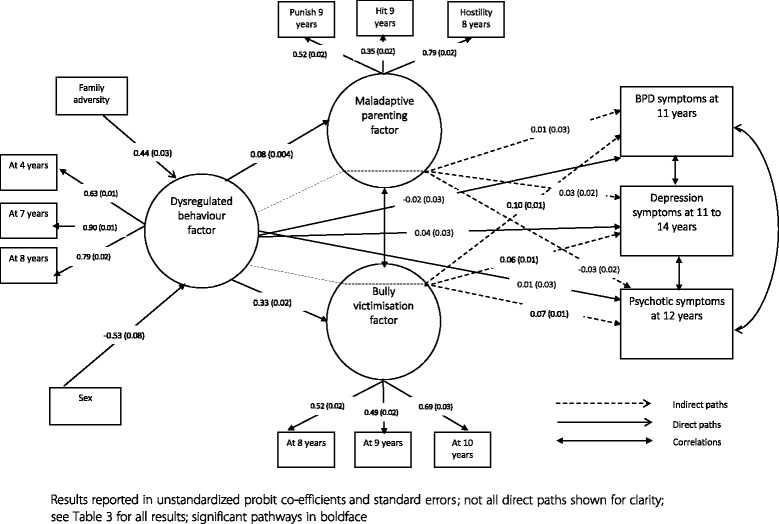
Final path model with main direct and indirect associations shown

Next, we modelled several simultaneous pathways to test our hypotheses (our final model is presented in Fig. 1). We modelled direct pathways from parenting and bullying factors to BPD, depression, and psychotic symptoms outcomes, and from sex and family adversity to childhood dysregulated behaviour and psychopathological (i.e., BPD, depression, and psychotic symptoms) outcomes. We modelled indirect associations between childhood dysregulated behaviour and BPD, depression, and psychotic symptoms outcomes via maladaptive parenting and bully victimisation. We also modelled correlations between parenting and bully victimisation factors, and the three psychopathological outcomes.

### Difference tests to examine the strength of the indirect associations between childhood dysregulated behaviour, environmental risks and BPD outcome versus depression and psychotic symptoms outcomes

The test the comparative strength of the indirect associations between childhood dysregulated behaviour via environmental risk to BPD outcome versus depression and psychotic symptom outcomes, we utilised an approach outlined in Lau and Cheung [48]. This method allows the strength of two specific mediated associations to be compared using the MODEL CONSTRAINT and DIFF TEST commands in *Mplus*. The test yields a difference score by subtracting one path coefficient from the other, and provides a significance value for this difference.

## Results

A total of 7.3% of the sample had 5 or more repeated or frequent BPD symptoms.

Unadjusted associations between childhood dysregulated behaviour, family adversity, maladaptive parenting measures (i.e., maternal hitting, punishment and hostility), and mother and child reported bully victimisation are reported in Table 2. Each of the individual variables were significantly associated with 5 or more BPD symptoms at 11 years.

**Table 2:** Unadjusted associations between childhood dysregulated behaviour, environmental risk factors, and subsequent BPD

Risk factor	BPD outcome
	Odds ratio(95% Confidence intervals)
Family adversity during pregnancy	**1.24 (1.17, 1.31)**
Maternal hitting	**1.41 (1.21, 1.65)**
Maternal punishment	**1.23 (1.11, 1.36)**
Maternal hostility	(no items reported)
One item	**1.72 (1.29, 2.29)**
Two items	**1.80 (1.33, 2.45)**
Three items	**2.22 (1.59, 3.11)**
Child reported bully victimisation at 8 years	**1.14 (1.12, 1.17)**
Mother reported bully victimisation at 9 years	(no bullying reported)
Somewhat true	**2.43 (1.90, 3.09)**
Certainly true	**4.67 (2.99, 7.29)**
Child reported bully victimisation at 10 years	**1.24 (1.21, 1.27)**
Dysregulated behaviour at 4 years	**1.08 (1.05, 1.11)**
Dysregulated behaviour at 7 years	**1.09 (1.06, 1.11)**
Dysregulated behaviour at 8 years	**1.09 (1.06, 1.12)**

Boldface indicates a significant association

### Confirmatory factor analysis

Our confirmatory factor analysis model including the three latent variables (i.e., dysregulated behaviour, maladaptive parenting, and bully victimisation) indicated adequate (TLI = 0.93) to good (CFI = 0.96; RMSEA = 0.05) model fit. All factor loadings for the latent variables were significant and of strong magnitude, ranging from 0.35 to 0.90 [49].

### SEM to examine the direct and indirect (via environmental risks) pathways from childhood dysregulation to BPD symptoms

A path model was specified to ascertain whether the association between childhood dysregulation and BPD was mediated by environmental risk factors. Fit indices indicated an acceptable (TLI = 0.93) to good (CFI = 0.96; RMSEA = 0.04) model fit. Direct associations between predictors and outcomes are reported in Table 3 (please also see Fig. 1 for the results of the final model). Male sex and family adversity were significantly associated with subsequent dysregulated behaviour. Dysregulated behaviour was associated with subsequent maladaptive parenting, bully victimisation, and depression. Indirect associations are reported in Table 4. There was a significant indirect association between childhood dysregulated behaviour and BPD (and depression and psychotic) symptoms via bully victimisation. There was no significant indirect association via maladaptive parenting for any of the psychopathological (i.e., BPD, depression, or psychotic symptoms) outcomes. The strength of indirect association between childhood dysregulation and BPD outcome via bully victimisation was significantly stronger than for the depression (Difference, BPD – depression symptoms: 0.034, *p* < 0.01) or psychotic symptoms (Difference, BPD – psychotic symptoms: 0.023, *p* < 0.01) outcomes.

**Table 3:** Path analysis results of direct associations between sex, family adversity, childhood dysregulated behaviour, maladaptive parenting, bullying victimisation, BPD, depression, and psychotic symptoms

Predictor	Outcome	Probit coefficient	SE	*P* value
Sex^a^	Dysregulated behaviour	**−0.527**	**0.075**	**<0.001**
Sex	Bully victimisation	−0.097	0.075	0.196
Sex	Maladaptive parenting	−0.010	0.008	0.192
Sex	Depression symptoms	**0.235**	**0.045**	**<0.001**
Sex	Psychotic symptoms	**0.110**	**0.047**	**<0.05**
Family adversity	Dysregulated behaviour	**0.440**	**0.027**	**<0.001**
Family adversity	BPD	**0.056**	**0.019**	**<0.01**
Family adversity	Depression symptoms	**0.056**	**0.016**	**<0.01**
Family adversity	Psychotic symptoms	**0.051**	**0.017**	**<0.01**
Dysregulated behaviour	Maladaptive parenting	**0.075**	**0.004**	**<0.001**
Dysregulated behaviour	Bully victimisation	**0.327**	**0.021**	**<0.001**
Dysregulated behaviour	BPD symptoms	−0.022	0.030	0.475
Dysregulated behaviour	Depression symptoms	0.036	0.025	0.147
Dysregulated behaviour	Psychotic symptoms	0.013	0.027	0.632

Probit coefficients indicate the strength of association between predictor variables and the probability of group membership, and represent the difference that a one-unit change in the predictor variable makes in the cumulative normal probability of the outcome variable

*SE* standard error; boldface indicates a significant association

^a^Male sex was reference category, thus significant negative co-efficient indicates that male sex is significantly associated with the outcome, while significant positive co-efficient indicates that female sex is significantly associated with the outcome

**Table 4:** Path analysis results of indirect associations between dysregulated behaviour and BPD, depression, and psychotic outcomes via maladaptive parenting and bully victimisation factors

	BPD symptoms			Depression symptoms			Psychotic symptoms		
	Probit co-efficient	SE	*P* value	Probit co-efficient	SE	*P* value	Probit co-efficient	SE	*P* value
Via maladaptive parenting	0.007	0.026	0.784	0.027	0.023	0.229	−0.032	0.023	0.172
Via bully victimisation	**0.097**	**0.009**	**<0.001**	**0.063**	**0.007**	**<0.001**	**0.074**	**0.008**	**<0.001**

Probit coefficients indicate the strength of association between predictor variables and the probability of group membership, and represent the difference that a one-unit change in the predictor variable makes in the cumulative normal probability of the outcome variable

*SE* standard error; boldface indicates a significant association

## Discussion

Our analyses indicated a significant indirect association between childhood dysregulation and BPD via an increased risk of bully victimisation (but not maladaptive parenting). A direct significant association between bully victimisation and BPD symptoms was also observed. While bully victimisation has been linked to a multitude of negative mental health outcomes [50, 51], this study reveals a prospective link between bully victimisation and BPD specifically, adding to an emerging evidence base highlighting the importance of peer relationships in the development of BPD [23, 15].

Both bully victimisation and maladaptive parenting were predicted by childhood dysregulated behaviour, suggesting that children evincing dysregulated behaviour from 4 to 8 years are more likely to attract negative attention from peers and parents. Studies have shown that childhood dysregulation is related to parental stress and maladaptive parenting practices [13]. Similarly, the association between childhood dysregulation and bully victimisation is well documented, with higher levels of dysregulation causing children to become more likely targets of victimisation [14]. Indeed, dysregulated children may be prone to short-temperedness, restlessness, and a tendency to retaliate when attacked. Further, they are prone to low self-esteem and social competence, coupled with high levels of aggression [53]. Consequently, once victimised, this pattern tends to persist for months or years even once the child changes school [54].

Bully victimisation rather than maladaptive parenting mediated the relationship between childhood dysregulated behaviour and BPD symptoms. There are two mechanisms via which this combined effect may occur, likely working in conjunction. First, bully victimisation may lead to the formation of negative relational schemata, altered social cognition, and a tendency to *hypermentalise* [55]. Hypermentalization - a propensity to over attribute other’s intentions - has been commonly observed in youths with BPD and may interact with dysregulation, preventing the development of healthy mentalising strategies [55]. While, negative biases are also found in association with psychosis and depression, they appear to be especially severe for BPD following exclusion and bullying [56]. In this way, an individual evincing dysregulated behaviour and encountering repeated negative interactions with others may develop maladaptive social strategies or “emotionally labile patterns of interaction” [5], manifesting as the core relational symptoms of BPD.

Second, for individuals evincing dysregulation, increased social stress due to victimisation may physiologically “work itself under the skin,” altering an already vulnerable stress response [57], potentiating emotional dysregulation and leading to further behavioural under control, manifesting as the core impulsive symptoms of BPD [5]. A vicious cycle may develop in which dysregulation is heightened, attracting more negative interactions, increasing dysregulation further, until trait dysregulation crystallises eventually culminating in a “borderline” personality [5].

It is surprising that maladaptive parenting did not mediate the association between dysregulation and BPD symptoms. These findings contrast with recent studies reporting an interaction between inherited diathesis, harsh parenting and subsequent BPD symptoms [17, 19]. It may be, however, that the aetiological impacts of parenting, excepting profound continuous abuse, are most influential early in childhood through the initiation of a chain of events [58], and are mediated by other factors later in the developmental trajectory [17]. Conversely, peer interactions may become especially salient as the child approaches adolescence and begins to spend more time with peers [20]. Another possible explanation is that shared method variance could have accounted for the especially strong association between bully victimisation and BPD symptoms (i.e., BPD and two of the bully indices were reported by the child). It should be noted, however, that mother-reported bullying was also strongly associated with BPD symptoms in the unadjusted analysis.

As bully victimisation adversely affects cognition, emotions and stress regulation, it is not surprising that depression and psychotic symptoms were also associated with childhood dysregulation via bully victimisation, though to a lesser extent than BPD symptoms. This may partly reflect symptom overlap and co-morbidity between disorders [59]. While the observed indirect relationship from childhood dysregulated behaviour via bully victimisation was significantly stronger for the BPD compared to the depression and psychotic symptoms outcomes, it was not unique to BPD. Future studies should seek to uncover aetiological pathways specific to BPD versus depression and psychotic symptoms.

Strengths of this study include the prospective longitudinal design, which eliminated problems associated with retrospective reports [60] and facilitated the use of path models to delineate risk trajectories to BPD symptoms in early adolescence. By utilising a large community sample (>4000 participants) we could consider how BPD symptoms may unfold in the general population, and incorporate an assessment of BPD which is comparable in composition to DSM diagnosis (i.e., 5 or more probable/definite symptoms).

Our study also has several limitations. Although we used a reliable assessment of BPD for children and adolescents [61], with comparable criteria to the adult diagnosis, we do not currently know what proportion of children evincing BPD symptoms at age 11 will develop BPD in adulthood. We will need to follow up these children into adulthood to determine how well the UK-CI-BPD predicts BPD. Nevertheless, previous research indicates that BPD symptoms in mid-adolescence predict BPD diagnosis in mid adulthood [62]. Second, there was substantial attrition in this study. Despite selective dropout we found strong and hypothesised associations between predictors and BPD symptoms among the remaining, less severely disadvantaged individuals. Previous simulations [52] have demonstrated that even when dropout is correlated to predictor/ confounder variables, the relationship between predictors and outcome is unlikely to be substantially altered by selective dropout processes. However, it cannot be precluded that dropout had some influence on the predictive relationships reported. Third, the extent to which the childhood dysregulated phenotype represents an independent risk factor for BPD requires further explication. It could be that the association between childhood dysregulation and BPD may have been partly attributable to an overlap between these two constructs. However, there was no direct association between these two constructs within the final path model, but a strong association via the experience of subsequent bully victimisation. Further, despite the comorbidity between BPD and other disorders, recent factor analytical studies support that BPD criteria are not fully accounted for by internalising and externalising psychopathology [63]. Finally, although we included a number of salient risk variables in our analysis, other unexplored factors, e.g., substance abuse, poor attachment relationships, and neglect [7], may have had an impact on the dysregulation and BPD measures.

## Conclusions

Children demonstrating higher levels of childhood dysregulation are prone to the development of BPD symptoms when exposed to environmental risk factors. Furthermore, dysregulated children are more likely to be exposed to these environmental risks. Therefore, effective interventions should target dysregulation early on in development [64] to reduce exposure to environmental risks and the canalisation of mental disorder [5]. The results of our study expand the existing literature by revealing the importance of peer relationships in the development of BPD symptoms, supporting that dysregulated victims are especially at-risk of negative sequalae. Pathways to BPD symptoms for those with dysregulated behaviour may be altered by interventions that reduce bully victimisation.

## Abbreviations

ALSAPC: Avon Longitudinal Study of Parents and Children
BPD: Borderline Personality Disorder
CFI: Comparative Fit Index
DSM: Diagnostic statistical manual
RMSEA: Root Mean Square Error of Approximation
SDQ: Strengths and Difficulties Questionnaire
SMFQ: Short Moods and Feelings Questionnaire
